# Quality improvement of clinic flow for complex genetic conditions: Using Ehlers–Danlos syndrome as a model

**DOI:** 10.1002/mgg3.472

**Published:** 2018-09-26

**Authors:** Preeti Prakash, Tanya N. Eble, Shweta U. Dhar

**Affiliations:** ^1^ Office of BCM Students Baylor College of Medicine Houston Texas; ^2^ Department of Molecular and Human Genetics Baylor College of Medicine Houston Texas; ^3^ Department of Internal Medicine Baylor College of Medicine Houston Texas

**Keywords:** adult genetics, clinic flow, Ehlers–Danlos syndrome, patient resources, quality improvement

## Abstract

**Background:**

Genetic providers face the challenge of having adequate time to conduct a comprehensive evaluation. Hypermobile Ehlers–Danlos (hEDS) syndrome has a complex array of symptoms. An initial visit can involve approximately 60–80 min and an additional 45 min for the check‐in and checkout process. We propose a model to improve clinic flow and patient satisfaction by using: (a) pre‐appointment questionnaire (b) disease information sheet outlining basic management and (c) itinerary detailing the visit.

**Methods:**

New patients were given a questionnaire, an EDS information sheet, and a visit itinerary. In the end, a patient satisfaction survey was administered containing 18 questions pertaining to their satisfaction with the questionnaire, the information sheet, and their overall visit. Completed surveys were turned in to the front desk to maintain anonymity.

**Results:**

Based on the survey results, patient satisfaction toward the implementation of a questionnaire was overwhelmingly positive. Survey responders found that the itinerary was added to their understanding of the appointment process and that the hEDS information sheets were helpful, understandable, and appropriate in length. Respondents said that they strongly agreed or agreed with the following statements: (a) I was satisfied with the visit; (b) I now have a better understanding of my condition; (c) This visit was successful in addressing my most pressing concerns; and (d) I would recommend this clinic to others.

**Conclusion:**

Designing a disease‐centered model that implements patient‐centered resources improves patient understanding and satisfaction for new hEDS patient visits. This model can be emulated in diagnosis and management of other complex genetic and nongenetic conditions.

## 1 INTRODUCTION

Specialty clinics that focus on genetic evaluation of adults are unique healthcare environments. Though many genetic conditions are diagnosed in childhood, there are many others that are diagnosed in adulthood and require follow‐up throughout life. As a result, adult genetics clinics are often inundated with patient appointments (Eble et al., 2012). Many of these patient appointments are for new diagnoses that require careful evaluation, as genetic conditions are often complicated in their manifestation. Hence, the evaluation of new genetic patients becomes challenging particularly within the current constraints of academic clinical practices where wRVUs measure a clinician's productivity and patient visits need to be completed within a specified time frame (Mezrich & Nagy, 2007).

Implementing quality improvement measures in healthcare settings is essential to providing accurate, efficient, and satisfactory care to patients. A variety of strategies has been implemented to assess patients with complex chronic conditions with the purpose of improving the quality and flow of patient appointments (Jones, Price, & Molen, 2011). However, there is a lack of information regarding effective methods of achieving this goal in a specialty adult genetics clinic. One study focused on reducing physician wait times by implementing a care coordinator to walk a patient through the process from referral to discharge and having patients fill out surveys detailing the care they desire prior to attending their appointment (Sampalli, Desy, Dhir, Edwards, & Dickson, 2015). Several centers have implemented their own measures to address the time‐consuming and complex array of symptoms exhibited by their patients. One such example involved requiring patients to complete a 31‐page previsit questionnaire prior to scheduling the initial consultation. Returning patients must also complete a follow‐up questionnaire if they have not visited the clinic in three or more years (Adult Genetics, 2017).

With these strategies in mind, the goal of our study was to implement a set of quality improvement measures in our Ehlers–Danlos Syndrome (EDS) Genetics Clinic focusing on new patient appointments for a potential diagnosis of EDS. EDS comprises a spectrum of hereditary connective tissue disorders that involve defects in the synthesis and functioning of collagen. These disorders are fairly common, with an overall prevalence of EDS of 1/5,000, resulting in an increasing demand for genetic evaluation for these conditions (Pauker & Stoler, 2016). Diagnosis of the most prevalent form of hypermobile EDS (hEDS) is challenging due to the multitude of symptoms associated with this disease, in addition to lack of confirmatory genetic testing (Malfait et al., 2017). The encompassing nature of this disease inevitably creates a large list of differential diagnoses that require careful evaluation to reach a conclusive diagnosis. With the limited amount of time available per appointment, it is difficult for patients to fully express their disease burden and concerns, while the physician is gathering information necessary to make a diagnosis. This in turn impacts the number of new hEDS patients that can be seen in the clinic per month, which ultimately leads to longer wait times for appointments, an increased risk of patients experiencing medical complications while waiting to be evaluated, and the potential for patient dissatisfaction (HealthFirst, 2017).

In the EDS Genetics Clinic, we were able to see 29 new patients for EDS evaluation during a 14 clinic‐day period from September 12, 2014, to November 6, 2014. Due to the complexity of a patient seeking evaluation for EDS and the time it took to review their records and manage care, we evaluated two new patients for EDS in a given clinic. Each evaluation often exceeded 120 min with the physician. This is as compared to a typical 60‐min new appointment in our general genetics clinic.

We implemented several strategies to optimize the clinic flow, increase the number of patients that could be accommodated, and improve patient satisfaction for new hEDS patient appointments, as patient satisfaction is an important parameter in evaluating the quality of a service (Gupta & Rokade, 2016). We incorporated a pre‐appointment questionnaire that elicits information about hEDS symptoms in an organized manner in order to guide geneticists in their history gathering, presentation, and assessment of a potential hEDS patient. Since these visits tend to be long, we gave an itinerary to all new patients that outlined their EDS clinic visit and helped to set expectations. An hEDS information sheet along with general management guidelines was also given to the patients during the visit so that they could review it and ask questions while still in clinic. This information sheet was given prior to the physical examination and full review of medical history with the caveat that a diagnosis had not yet been made. We hypothesized that use of a pre‐visit questionnaire, itinerary, and information sheets particularly for chronic conditions such as hEDS would not only allow a physician to ascertain all of the pertinent information necessary to form a diagnosis in a time‐effective manner, but also improve patient satisfaction for new patient visits and decrease the amount of time necessary for obtaining a medical history.

## 2 MATERIALS AND METHODS

### 2.1 Ethical compliance

The Institutional Review Board that includes an ethics committee approved this proposal.

**Figure 1:**
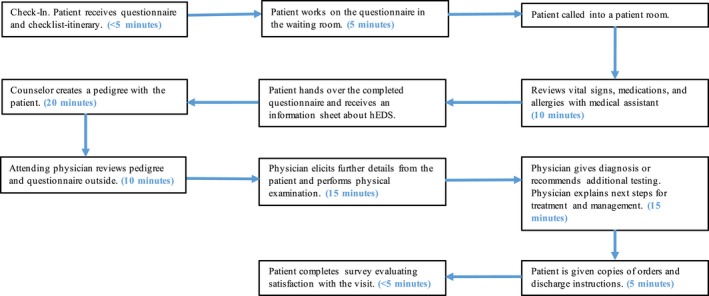
Flowchart for a new hEDS patient visit: The appointment flow begins at the check‐in desk in the waiting room. The patient receives a new patient questionnaire and a checklist itinerary that outlines each step of the appointment. Once their initial information and vital signs are recorded, a resident/medical student trainee or genetic counselor obtains family history information to create a pedigree. At this time, the patients are provided an information sheet about hEDS to review while they wait for the attending physician, with the caveat that no diagnosis has yet been made. In the interim, the attending physician reviews the pedigree and completed questionnaire outside the examination room. The physician can then focus on the pertinent information learned from the questionnaire and elicit further details from the patient as necessary. The physician conducts a physical examination and, based on the composite information, makes a clinical diagnosis or recommends additional testing. The assessment and plan for the patient's care are explained in detail, with instructions for further evaluation provided. To conclude the visit, the patient fills out a survey to evaluate his or her satisfaction with the visit

The Baylor College of Medicine Genetics Clinic dedicates one day per month for the evaluation of new adult patients with a potential diagnosis of EDS. Most patients are self‐referred and generally present with symptoms such as joint pain or dysautonomia. Figure 1 outlines the flow of the clinic appointment in detail. With the implementation of the pre‐appointment questionnaire and the EDS information sheet, we planned each new patient visit from check‐in to completion of the survey to be around 60 min, but physician appointments were scheduled 30 min apart. No additional genetic counseling or staffing was required by the implementation of the pre‐appointment questionnaire and information sheet. We were able to schedule visits 30 min apart because detailed information regarding medical history was obtained through the pre‐appointment questionnaire. Genetic counselors obtained the family history information, drew the pedigree, and provided discharge information to the patients. Thus, we were able to overlap patients, schedule in 30‐min intervals based on the face‐to‐face time the physician required with the patient to conduct the physical examination and discuss the diagnosis and plan for management.

**Figure 2:**
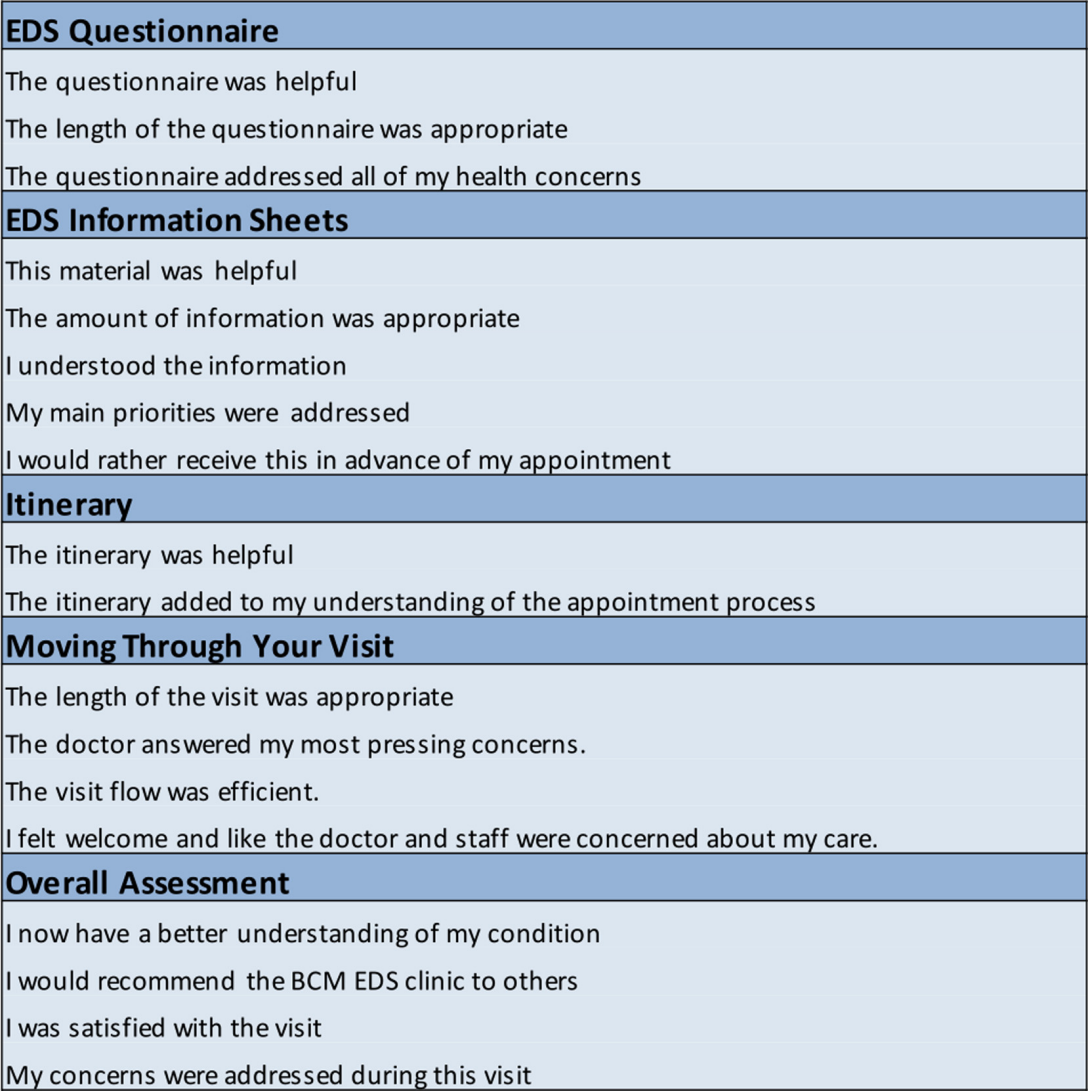
EDS Questionnaire: A list of the 18 questions asked in the EDS new patient feedback survey. We elicited feedback on the pre‐appointment questionnaire, information sheets, itinerary, the visit experience, and the patient's overall assessment of the appointment. For each question, the patient checked off one of the following sentiments: Strongly Agree, Agree, Neutral, Disagree, and Strongly Disagree

An itinerary was given to patients at check‐in. It provided a checklist of each step of the new EDS patient visit process. The pre‐appointment questionnaire included questions about the patient's present illness, past medical history, and a thorough review of systems. Aside from generalized questions about a patient's health history, the questionnaire asked pertinent questions specific to the manifestation of hEDS. These questions were based on the characterization of hEDS illustrated in the literature as well as the authors’ clinical experience with hEDS patients as a geneticist (Castori et al., 2017). Prior to the appointment, the patient received the questionnaire in the mail; however, we noted that the majority of patients did not come to clinic with the completed questionnaire, but rather filled it out on the day of the appointment either in the waiting room or in the examination room while they were waiting to be seen. The information sheets given in the patient room included information about the diagnosis, inheritance, and management of EDS as well as frequently asked questions. Patients were provided these information sheets prior to receiving a diagnosis by the clinical geneticist to address commonly asked questions about EDS. To conclude the visit, the patient filled out a survey to evaluate his or her satisfaction with the visit. The 18‐question survey assessed patient opinion on the EDS questionnaire, information sheets, itinerary, moving through the visit, and on the appointment overall. There was space at the end of the survey for patients to freewrite additional positive or negative comments about the visit. Figure 2 summarizes the specific questions asked in the survey. If a patient did not have hEDS, they were discharged from clinic with that information, and if a patient needed further investigation for more complex disorders, a follow‐up appointment was scheduled.

## 3 RESULTS

**Figure 3:**
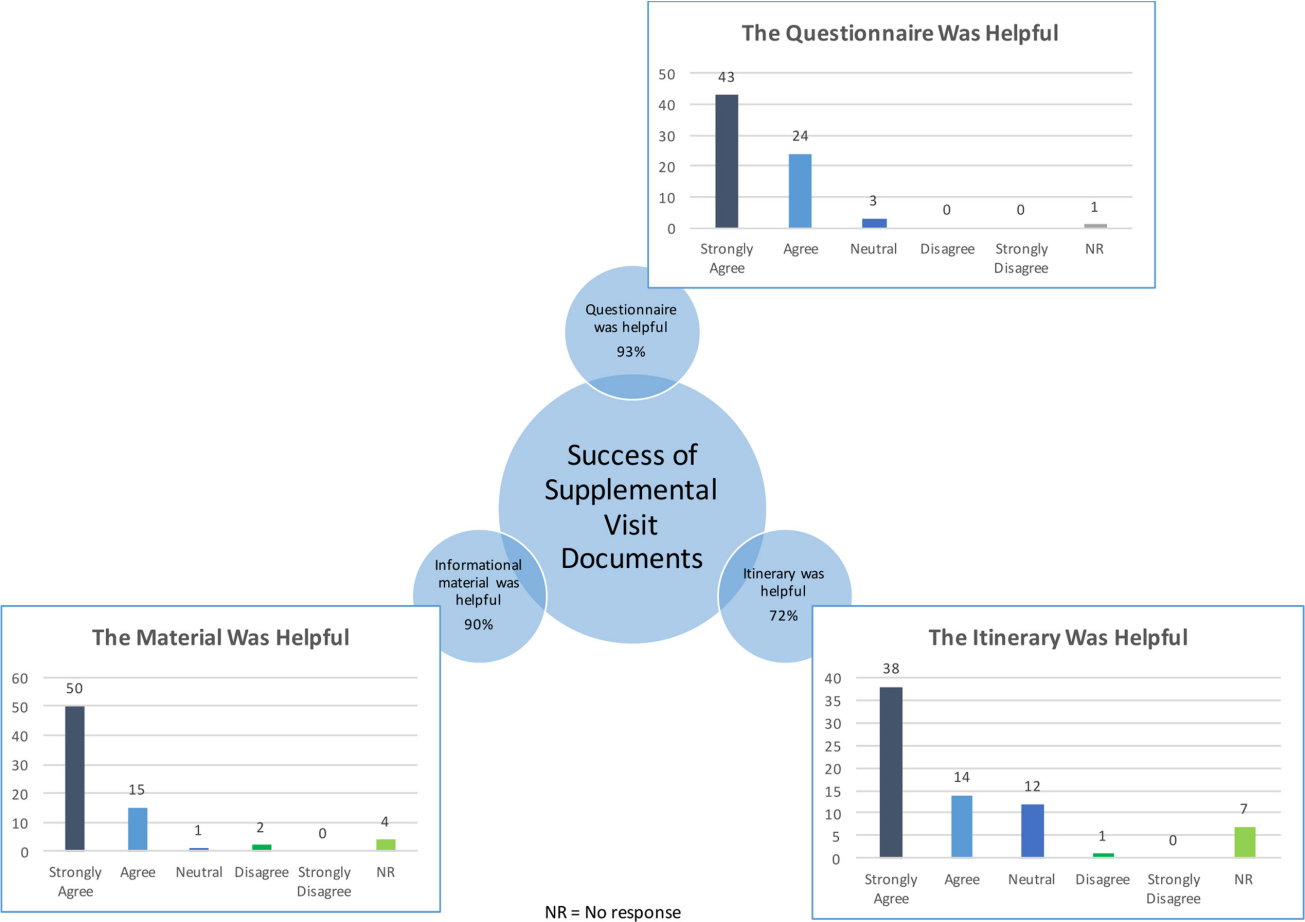
Success of supplemental visit documents: This figure summarizes the success of using the supplemental documents that were distributed during the EDS new patient appointment and how they compare to each other. Based on our findings, clinics may find the most improvement in using the questionnaire and the informational material

Between the months of November 2014 and May 2016, a time period encompassing 14 EDS clinic days, 72 out of 94 patients who attended an EDS new patient visit completed and returned the survey for a total response rate of 77%. With regard to the pre‐appointment questionnaire, 93% (*n* = 67) of patients found the questionnaire to be helpful. Ninety percent (*n* = 65) of patients responded positively toward the questionnaire with regard to its length, and 85% (*n* = 61) responded positively toward its ability to address the patient's health concerns. Of note, 4% (*n* = 3) did not believe the questionnaire addressed all of their health concerns (Figure 3).

With regard to the information sheets provided during the appointment, 90% (*n* = 65) of patients found the EDS information sheets to be helpful and 94% (*n* = 68) found them understandable. Ninety percent (*n* = 65) of patients felt that the amount of information presented in the sheets was appropriate. A majority of patients, 86% (*n* = 62), believed their main priorities were addressed with the information sheets, and 8% (*n* = 6) patients felt that the sheets did not expound on their main priorities. While 40% (*n* = 29) of patients responded that they would rather receive the information sheets prior to their appointment, 15% (*n* = 11) disagreed or strongly disagreed with this statement (Figure 3).

**Figure 4:**
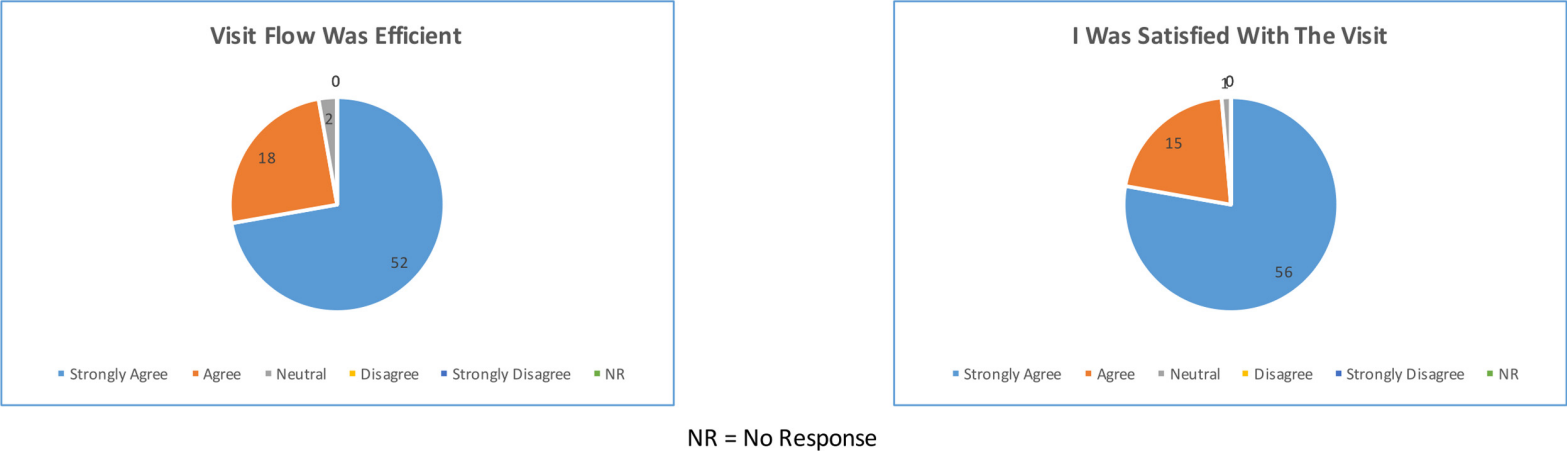
Overall patient assessment of EDS clinic: A summary of patient survey responses in terms of their satisfaction with the visit and overall efficiency of the clinic flow. Ninety‐seven percent of patients found the visit flow to be efficient, and 99% of patients were satisfied with the visit

In terms of the clinic experience, about 72% (*n* = 52) of patients found the itinerary to be helpful and 75% (*n* = 54) of patients found that the itinerary broadened their understanding of the appointment process (Figure 3). The length of the clinic visit from check‐in to completion of the survey was determined to be around 90 min. As patients reflected on moving through their visit, 96% (*n* = 69) of patients responded favorably to the length of the visit and 97% (*n* = 70) of patients found the visit to be efficient. By the end of the clinic appointment, 99% (*n* = 71) of patients felt that the physician answered their most pressing concerns. Overall, 99% (*n* = 71) of patients were satisfied with their visit and 97% (*n* = 70) of the surveyed patients would recommend the Baylor College of Medicine EDS Genetics Clinic to others based on their experience (Figure 4).

The comments that patients entered in the survey were predominantly positive. Notable comments included, “Thank you for taking your time in listening to our concerns and answering questions. Thank you for the literature. It is helpful” and “Thank you! First time someone took the time with me to listen to my history and concerns‐ 41 years.” Constructive criticism about the new patient process concerned patients feeling rushed or unprepared during the questionnaire‐filling process. Examples of the constructive comments include “I appreciate not waiting but felt rushed in doing my new patient paperwork and wasn't informed if it was online or not” and “Remind patient about paperwork/survey.”

During the 14 clinic days in which our strategies to increase EDS patient volume and satisfaction were implemented, a total of 94 new patients were seen for EDS evaluation as compared to 29 new patients seen for EDS evaluation during the 14 clinics prior to implementation of the itinerary, questionnaire, and information sheet. Thus, we were able to increase our new EDS patient volume from two patients per clinic to seven patients per clinic (350% increase).

## 4 DISCUSSION

Hypermobile Ehlers–Danlos syndrome (hEDS) continues to be a unique diagnostic challenge for health providers due to its complex array of symptoms such as joint hypermobility, skin hyperelasticity, chronic musculoskeletal pain, and dysautonomia, which are variably expressed among patients and somewhat nonspecific. Also, the underlying genetic etiology of hEDS is still unknown necessitating the diagnosis to be entirely clinical.

There is a growing need to focus on patient satisfaction and to ensure that patients’ concerns are heard and addressed in the current medical practice milieu. Several health institutions have implemented Press Ganey surveys for patients to complete after their clinic and hospital experiences, and the data collected from these surveys have ultimately driven quality improvement measures addressing patient satisfaction (Patient Satisfaction Surveys ‐ Press Ganey, 2017). Recognizing that many specialties are now focusing on clinic efficiency and patient satisfaction, we sought to explore methods of quality improvement in our EDS clinic. We created a questionnaire, itinerary, and information sheets to guide health providers and patients through an hEDS diagnosis. We then evaluated the quality of our improvement measures by implementing a patient survey to assess patient satisfaction. This evaluation was necessary because, while our institution does participate in Press Ganey data collection, the data for the various genetics subspecialty clinics are available only in aggregate and it was not possible to obtain information specific to the EDS clinic. To remedy the lack of satisfaction data specific to the population of patients seeking evaluation for EDS and related to our strategies of quality improvement, we created a survey.

It is well known that questionnaires are a standardized, structured method of assessment that can cover a plethora of symptoms involved in a disease. Patient‐reported questionnaires can highlight the patients’ perception of their disease and quality of life beyond the objective measures physicians use to assess disease severity. From the questionnaire, physicians can determine the issues most pertinent to the patient, which ultimately facilitates a patient–physician discussion based on mutual understanding of the patient's symptoms and concerns. Patient‐reported questionnaires provide physicians with a holistic perspective on the impact of a patient's condition, which can direct the flow of the appointment toward a targeted diagnosis, intervention, and management. We found that pre‐appointment questionnaires can ultimately create reasonably timed, focused appointments for patients with complex chronic conditions that are not only thorough, but also patient‐centered. We were able to decrease the scheduled physician time with patients from 60‐min appointment slots in the 14 clinic‐day period prior to implementation of the improvement strategies to 30‐min appointment slots in the 14 clinic‐day period after implementation of the improvement strategies. This greater clinic efficiency also enabled the evaluation for EDS of, on average, seven new patients per clinic as compared to two patients prior to implementation of these strategies.

Based on the data, patient satisfaction toward the implementation of a questionnaire in new clinic visits was overwhelmingly positive (85% patients felt that the questionnaire was able to address their health concerns effectively). Patients felt that the questionnaire was helpful, of an appropriate length, and adequately allowed them to express their health concerns. Because the questionnaire covers the plethora of systems affected by EDS, it relieves patient burden regarding remembering to discuss each aspect of their experience with the disease and can allow for visit time to elaborate on the most pressing concerns. A small percentage (4%) of patients felt that the questionnaire did not fully address their health concerns. Though the questionnaire provides a framework for patients to state their health concerns, any issue that is not listed in the questionnaire can be addressed directly to the physician during the clinic visit. This is evidenced by the data showing that 99% of patients felt that their concerns were ultimately addressed by the conclusion of the visit. Based on the free‐written survey comments concerning the time needed to fill out the questionnaire, perhaps it would be beneficial to remind patients to arrive 15–30 min prior to their appointment in order to have adequate time to fill out the questionnaire in a relaxed manner. Alternatively, patients could be required to submit their completed questionnaire prior to scheduling an appointment. Overall, the questionnaire provides patients a platform to expound on the many symptoms of hEDS and present a clear clinical picture to a geneticist in an organized, thorough manner. Such a questionnaire could be developed for other complex conditions or more broadly for a range of conditions commonly seen in a given specialty clinic. The physician providers using this questionnaire in our clinic found it to be very helpful in coming to a diagnosis as all the information was available in an organized and efficient manner. It also shortened the time of history taking particularly since patients with hEDS tend to have complex and lengthy histories.

Moreover, the survey responders found the EDS information sheets to be helpful, understandable, and appropriate in content. A majority of patients found that the information addressed their main priorities concerning their illness. For the 8% of patients who felt that the information sheets did not address their priorities, we argue that the information sheets were meant to provide an outline of the condition and its management but not to replace counseling and physician education for hEDS. Any additional concerns could be brought up directly to the physician. A number of responders (40%) would rather have read this information prior to their clinic appointment. It might be contended that the information and FAQ sheets could be provided prior to the appointment through mail or email, allowing patients to gain knowledge about their potential diagnosis prior to their appointment and prompt more in‐depth questions once they meet with their geneticist. However, there are distinct disadvantages to this approach. It might give the false impression to the patient that they already have a diagnosis of hEDS. It also has the potential to bias patient responses in the pre‐appointment questionnaire.

With regard to the visit itinerary, the majority of patients found this helpful. Patients felt that it added to their understanding of the appointment process. Itineraries or preplanned schedules have been traditionally used in other fields, such as the travel industry. Itineraries not only provide an outline on future steps in a process, but also aid in time management. Clinic visits for a new diagnosis can be lengthy and complicated. Providing a clear itinerary allows patients to be more involved and aware of the clinic process, which can ultimately help with the diagnostic evaluation.

The survey indicated that patients found their visit to be appropriate in length and efficient. Though the average clinic appointment time was 90 min, we did not document the actual clinic appointment length for patients in relation to their survey responses in order to preserve patient confidentiality. Thus, it is unclear if the patients who responded favorably to the length of the visit had a longer or shorter appointment time. We can conclude, however, that the implementation of our quality improvement measures did not negatively affect patient satisfaction in terms of the length of clinic appointments.

As an overall assessment, survey responders found the clinic visit to be successful in addressing their most pressing concerns. The majority of survey responders felt that they left their visit with a better understanding of their condition, were satisfied with their visit, and would recommend this clinic to others. The free‐written comments on the survey illustrate that patients felt that their struggles were heard and understood. Strong patient–physician relationships have been shown to increase patient compliance and improve clinical outcomes. When clinics place emphasis on showing care and concern for a patient's health, it improves patient satisfaction and inspires patients to have greater concern for their own health (Swaminath, 2007).

Limitations of our study concern the fact that the patient satisfaction surveys were anonymous, and therefore, responses could not be correlated with individual patient variables. Additionally, the survey did not ask whether a patient received a hEDS diagnosis during the visit. A patient's perspective of the clinic visit could be influenced by whether they received a diagnosis of hEDS. Some survey responders might have been seen for confirmation of a known diagnosis of hEDS. Additionally, though our response rate was high (77%), not all of the patients who presented for a new EDS clinic visit filled out the survey. Thus, our study might not account for the opinions of all patients who had a new visit in the EDS clinic during the study period.

Overall, this study highlights the promising nature of several strategies including a pre‐appointment questionnaire, clinic itinerary, and disease information sheets with management guidelines in improving patient understanding and satisfaction for new EDS patient visits. This systematic and organized approach in addressing the complexity of an hEDS evaluation could potentially be used for the diagnosis of other complex genetic and nongenetic conditions in new patient visits. In terms of future directions, this study focused on whether the process of using a pre‐appointment questionnaire improved patient satisfaction with new hEDS patient clinic visits. It would be vital to also analyze whether this process aids in improving the average time of new patient visits, as increased efficiency allows physicians to diagnose more patients on dedicated new patient visit days and would pose less of a time burden on patients.

## CONFLICT OF INTEREST

The other authors declare no conflict of interest.
